# Outcomes and longitudinal trend of traumatic cataract wound dehiscence in patients with blunt ocular injury

**DOI:** 10.1038/s41598-021-97723-4

**Published:** 2021-09-14

**Authors:** Chiun-Ho Hou, Yu-Chin Lu, Christy Pu, Yin-Hsi Chang, Ken-Kuo Lin, Jiahn-Shing Lee, Kuan-Jen Chen

**Affiliations:** 1 Department of Ophthalmology, Chang Gung Memorial Hospital, Linkou Medical Center, Taoyuan, Taiwan; 2 Institute of Public Health, School of Medicine, National Yang Ming University, Taipei, Taiwan; 3 Department of Ophthalmology, Change Gung Memorial Hospital, Xiamen, People’s Republic of China; 4 Department of Medicine, College of Medicine, Chang Gung University, Taoyuan, Taiwan

**Keywords:** Medical research, Risk factors

## Abstract

Longitudinal trends on traumatic cataract wound dehiscence are scant. In this study, we present the characteristics of traumatic cataract wound dehiscence using 15 years of longitudinal trend in one of the largest medical centers in Taiwan for a period when cataract surgeries were gradually shifting from extracapsular cataract extraction (ECCE) to phacoemulsification. All patients with a prior cataract surgery who suffered from blunt open globe trauma between 2001 and 2015 at a tertiary referral center in Taiwan were included. The number of cases per year; type of prior cataract surgery; visual acuity (VA); mechanism and place of injury were analyzed. The risk factors associated with final VA were investigated in patients followed up for ≥ 1 month. Seventy-six eyes of 75 patients were included and all of them were traumatic cataract wound dehiscence with a prior ECCE (65 eyes) or phacoemulsification. The most common mechanism and place of injury was fall and at home in both cataract surgical types. The mean log of the minimal angle resolution (logMAR) of final VA was 2.15 ± 0.88 (ECCE) and 1.61 ± 0.83 (phacoemulsification) (P = .026). The most significant risk factors associated with worse final VA were retinal detachment at the initial visit and low ocular trauma score (both P < .001). Long-term visual outcome of phacoemulsification wound dehiscence was better than that of ECCE wound after a blunt trauma.

## Introduction

Surgery remains the most effective treatment for cataract. Two major techniques are employed for performing cataract surgeries: manual extracapsular cataract extraction (ECCE) and phacoemulsification^1^. ECCE involves a scleral incision of 9 to 13 mm. Phacoemulsification is performed with a small scleral tunnel or a clear corneal incision, and the incision size is approximately 5 mm with a rigid intraocular lens (IOL) implantation and ≤ 3 mm with a foldable IOL implantation. Both ECCE and phacoemulsification involve creating a penetrating wound on the sclera or cornea that may act as a weak point against increased ocular pressure arising from a blunt ocular injury.

Traumatic cataract wound dehiscence after cataract surgeries is not uncommon^2,3^. Population-based surveys in the Unites States and Australia have demonstrated that patients undergoing phacoemulsification cataract surgery outnumbered those undergoing ECCE surgery after 1995^4,5^. Cases of traumatic phacoemulsification wound dehiscence have been reported since 1999^6–10^. However, these studies were either case reports or small case series. Studies that involve larger sample sizes with multiple time periods are lacking, and the comparison of the risk factors associated with poor visual outcome between ECCE and phacoemulsification is not yet available.

The present study investigated the outcome and longitudinal trend of traumatic cataract wound dehiscence in a tertiary referral medical center in a period when cataract surgeries were gradually shifting from ECCE to phacoemulsification. Longitudinal observations allow the determination of dynamic changes in occurrence and outcome. We also evaluated the visual outcome and risk factors associated with poor visual outcome after traumatic cataract wound dehiscence. To the best of our knowledge, this is the first study to demonstrate the longitudinal trend of traumatic cataract wound dehiscence and the comparison of risk factors between ECCE and phacoemulsification wound in visual outcome. Furthermore, our study investigated the largest number of patients with prior phacoemulsification, and the visual outcome for more than two months of follow-up was analyzed.

## Methods

The present study is a retrospective consecutive case series which involves medical record reviews for patients who experienced blunt open globe trauma after cataract surgery between January 2001 and December 2015 at a tertiary referral medical center in Northern Taiwan. The Institutional Review Board of Chang Gung Memorial Hospital in Linkou, Taiwan, approved the study protocol (IRB No. 104-9677B) and waived the requirement of written informed consent. All methods were carried out in accordance with relevant guidelines and regulations in the manuscript.

A blunt open globe trauma is defined as a full-thickness wound in the corneoscleral wall. Patients who experienced a blunt open globe trauma in the eye with a history of cataract surgery were included. We excluded patients with (1) penetrating wounds from sharp objects because wounds created by sharp objects do not have a pressure built up as in those from blunt trauma and (2) a history of previous penetrating keratoplasty or previous globe penetration injury because these wounds can be another weak area in the globe other than cataract wound.

Demographic characteristics; medical histories, including time, place, and mechanism of injury; ophthalmological examination; and image studies of these patients were collected. Emergency wound repair was performed in the absence of contraindications, and intravenous antibiotic was administered for 3 to 5 days accordingly. The visual acuity (VA) in the patient’s initial and follow-up visits was measured using the log of the minimal angle of resolution (logMAR)^11^. For patients with VA of < 0.05, we converted VA of finger counting, hand movement, only light perception, and no light perception into logMAR values of 2.0, 2.3, 2.6, and 2.9, respectively^12,13^. To analyze changes in visual outcomes after traumatic cataract wound dehiscence, we excluded patients who were followed up for < 1 month after trauma as visual outcomes were not likely to be stable within a short period.

We then analyzed the risk factors associated with final VA using ANOVA test and multiple linear regression. The risk factors included were initial VA, hyphema, vitreous hemorrhage, IOL location (in place, dislocation but inside the globe, partially outside the globe, or completely outside the globe), type of cataract surgery (ECCE or phacoemulsification), retinal detachment (RD) at the initial visit, globe deformity on imaging, and ocular trauma score (OTS, as Table 1). Chi-squared tests, t tests, and ANOVA tests were performed for demographic and clinical presentation analysis. Fisher’s exact tests were performed for categorical variables if any category had fewer than 5 patients. If a specific variable was not available for a patient, that patient was excluded from that particular analysis. A *P* value of < *0.0*5 was considered significant. All analyses were conducted using STATA 15^14^.

**Table 1:** The variables and raw points for computing Ocular Trauma Score (OTS).

Variables	Raw points
**Initial vision**	
NLP	60
LP/HM	70
1/200 to 19/200	80
20/200 to 20/50	90
≧20/40	100
Rupture	− 23
Endophthalmitis	− 17
Perforating injury	− 14
Retinal detachment	− 11
Afferent pupillary defect	− 10

*HM* hand motion, *LP* light perception, *NLP* no light perception.

## Results

This study included 76 eyes of 75 patients suffered from blunt open globe trauma and all of these wounds were traumatic cataract wound dehiscence. One patient had her right eye injured in 2012 and left eye in 2013. The demographic characteristics of these patients are presented in Table 2. This cohort had more women than men. The mean age of the patients was similar for male (75.1 ± 10.1 years) and female (74.7 ± 9.6 years) patients (P = 0.867). Overall, 65 and 11 eyes (86% and 14%) underwent ECCE and phacoemulsification, respectively. The most common mechanism of injury was fall (48 eyes, 63%), and the most common place of injury was at home (56, 74%). There is no statistical difference between ECCE and phacoemulsification groups in age, sex, and etiology and place of injury. Most patients sought medical help within 24 h after injury, and received surgical treatments within 24 h in emergency room. No significant difference was noted between male and female patients in etiology (*P* = 0.256) and place of injury (*P* = 0.071). Seventy-five percent (56 patients) of the included patients had a history of ≥ 1 chronic systemic diseases, with the most frequently observed being hypertension (20 patients, 36%), diabetes mellitus (13, 23%), chronic heart failure (5, 9%), and previous cerebral vascular accident (3, 5%).

**Table 2:** Demography and characteristics of patients with traumatic cataract wound dehiscence.

	Total, n (%)	ECCE, n (%)	PEA, n (%)	P value*
Number	76	65	11	
Age (years, mean ± SD)	74.8 ± 9.7	74.8 ± 9.9	76.27 ± 8.5	0.597
**Sex**				
Male	29 (38%)	23 (35.4%)	6 (54.5%)	0.226
Female	47 (62%)	42 (64.6%)	5 (45.5%)	
**Eye**				
Right	37 (49%)	30 (46.2%)	7 (63.6%)	0.283
Left	39 (51%)	35 (53.8%)	4 (36.4%)	
**Injury place**				
Home	56 (74%)	47 (72.3%)	9 (81.8%)	0.508
Outside home	20 (26%)	18 (27.7%)	2 (18.2%)	
**Injury etiology**				
Fall	48 (63%)	39 (60.0%)	9 (81.8%)	0.425
Hit by object	20 (26%)	18 (27.7%)	2 (18.2%)	
Assault	8 (11%)	8 (12.3%)	0 (0.0%)	
**Duration between trauma and visit**				
With 24 h	64 (84%)	53 (81.5%)	11 (100%)	0.589
24 to 48 h	9 (12%)	9 (13.8%)	0 (0.0%)	
> 48 h	3 (4%)	3 (4.6%)	0 (0.0%)	
**Duration between surgical repair and trauma**				
No repair	4 (4%)	4 (6.2%)	0 (0.0%)	1.000
< 24 h	67 (88%)	56 (86.2%)	11 (100%)	
24–48 h	5 (7%)	5 (7.7%)	0 (0.0%)	

*ECCE* extracapsular cataract extraction, *PEA* phacoemulsification.

*P value of statistical test between ECCE and PEA.

The clinical presentation after traumatic cataract wound dehiscence in these 76 eyes was listed in Table 3. Initial VA and OTS were both better in the phacoemulsification group than in the ECCE group. The percentage of hyphema, vitreous hemorrhage, IOL dislocation and RD at the initial visit was all higher in ECCE group. Sixty-three patients (83%) underwent ocular ultrasonography or computed tomography, and the percentage of globe deformity was higher in the ECCE group. Only two eyes with RD at the initial visit received pars plana vitrectomy combined with primary repair surgery. Final vision (logMAR) was 1 and 2.3, respectively. Three eyes with RD at the initial visit received pars plana vitrectomy with or without scleral buckling 1, 4, 7 weeks after primary repair, and final vision (logMAR) was 2.9, 2.3, and 2, respectively. Severe hemorrhagic suprachoroidal detachment was associated with RD at the initial visit in 37 (out of 42) eyes with RD. Because of poor visual prognosis, vitrectomy was not recommended. Three eyes developed RD after primary repair surgery. Pars plana vitrectomy with or without scleral buckling was performed 1, 2 and 10 weeks after primary repair surgery. Final vision (logMAR) was 2, 2, and 1, respectively. One patient in the ECCE group developed endophthalmitis. The infection subsided after intravitreous injection of antibiotics, and the final VA was no light perception. Four patients in the ECCE group did not receive surgical repair due to their physical condition, including 2 individuals who died from a heart attack or intracranial hemorrhage.

**Table 3:** Clinical presentation at the initial visit after traumatic cataract wound dehiscence in patients with prior extracapsular cataract extraction and phacoemulsification (all 76 eyes are included).

	All patients		ECCE		PEA		P value*
	n	(%)	n	(%)	n	(%)	
Vision at initial visit logMAR (mean ± SD)	76	2.50 ± 0.38	65	2.55 ± 0.37	11	2.21 ± 0.47	0.0049
**Hyphema**							
No	11	(14.5%)	6	(9.2%)	5	(45.5%)	0.002
Yes	65	(85.5%)	59	(90.8%)	6	(54.5%)	
**Vitreous hemorrhage**							
No	16	(21.1%)	10	(15.4%)	6	(54.5%)	0.003
Yes	60	(78.9%)	55	(84.6%)	5	(45.5%)	
**Intraocular lens position**							
In place	11	(14.5%)	8	(12.3%)	3	(27.2%)	0.010
Dislocation but inside the globe	4	(5.3%)	3	(4.6%)	1	(9.1%)	
Partially outside the globe	6	(7.9%)	3	(4.6%)	3	(27.3%)	
Completely outside the globe	55	(72.3%)	51	(78.5%)	4	(36.4%)	
**Retinal detachment**							
No	34	(44.7%)	25	(38.5%)	9	(81.8%)	0.009
Yes	42	(55.3%)	40	(61.5%)	2	(18.2%)	
**Globe deformity**							
No	26	(34.2%)	19	(29.2%)	7	(63.6%)	0.045
Yes	37	(48.7%)	35	(53.9%)	2	(18.2%)	
Not available	13	(17.1%)	11	(16.9%)	2	(18.2%)	
Ocular trauma score (mean ± SD)	76	31.1 ± 12.7	65	29.7 ± 12.4	11	39.5 ± 11.3	0.019

*ECCE* extracapsular cataract extraction, *PEA* phacoemulsification, *logMAR* log of minimal angle of resolution.

*P value of statistical test between ECCE and PEA.

**Figure 1:**
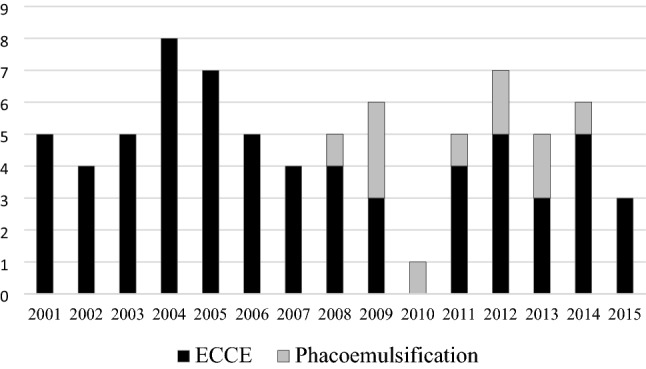
Number of eyes with traumatic cataract wound dehiscence with prior ECCE or phacoemulsification surgery from 2001 to 2015. *ECCE* extracapsular cataract extraction.

The patients with traumatic cataract wound dehiscence before 2008 all had a prior ECCE, and cases of a phacoemulsification wound appeared almost every year since the first case in 2008. Figure 1 shows the number of eyes in each year with traumatic cataract wound dehiscence with a prior cataract surgical technique from 2001 to 2015.

**Figure 2:**
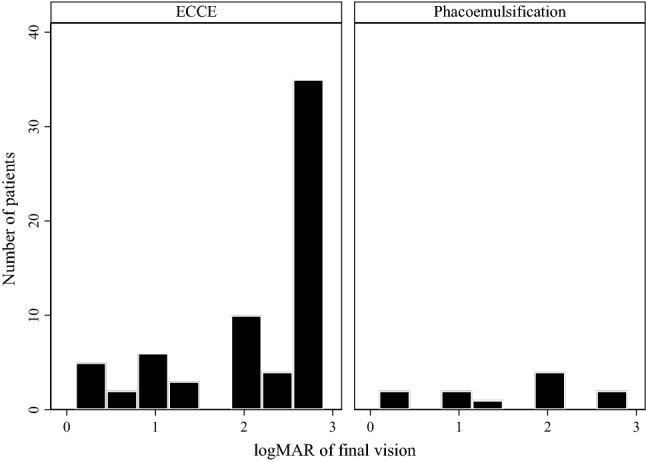
Final visual acuity of eyes after traumatic cataract wound dehiscence in ECCE and phacoemulsification groups. In cases of VA of < 0.05, finger counting at 2 feet, hand movement, only light perception, and no light perception were assigned logMAR values of 2.0, 2.3, 2.6, and 2.9, respectively. *ECCE* extracapsular cataract extraction.

Three patients in the ECCE group were followed up for < 1 month, including the 2 who died. These 3 cases were excluded from the VA analysis, and thus, 73 eyes were included. The minimum follow-up duration was 1 month in the ECCE group and 2 months in the phacoemulsification group. Fifty-one patients (82%) in the ECCE group and 8 (73%) in the phacoemulsification group were followed up for > 6 months. The mean logMAR of final VA was 2.07 ± 0.90 in these 73 eyes. The mean logMAR of final VA was 2.15 ± 0.88 in the ECCE group and 1.61 ± 0.83 in the phacoemulsification group (*P* = 0.026). The final VA in the ECCE and phacoemulsification groups is shown in Fig. 2. The percentage of final VA indicating no light perception was 40% (25/62) in the ECCE group, which was higher than the 9% (1/11) in the phacoemulsification group (*P* = 0.046). No significant difference was noted between final VA before 2008 and after (including) 2008 (*P* = 0.542).

ANOVA analysis for risk factors associated with worse final VA after traumatic cataract wound dehiscence were analyzed by ECCE and phacoemulsification groups, and the results are presented in Table 4. RD at the initial visit and low OTS are significantly associated with poor final VA for both ECCE group (p < 0.001; < 0.001) and phacoemulsification group (p = 0.022; 0.008). We also performed multiple linear regressions for these risk factors. RD at the initial visit was associated with poorer final VA in the regression model excluding OTS (p < 0.001). When OTS was included in the model, low OTS was associated with poorer final vision (p < 0.001).

**Table 4:** Risk factors analysis for final visual outcome (logMAR) by extracapsular cataract extraction (ECCE) and phacoemulsification

Demographic variables	ECCE				Phacoemulsification			
	n	Mean	SD	p-value	n	Mean	SD	p-value
**Age**	62			0.607	11			0.096
< 60	8	1.781	1.161		0	–	–	
60–70	11	2.075	0.694		3	1.366	0.552	
70–80	25	2.220	0.826		5	2.160	0.619	
> 80	18	2.255	0.953		3	0.932	0.925	
**Sex**	62			0.496	11			0.560
Female	39	2.207	0.793		5	1.780	0.634	
Male	23	2.047	1.028		6	1.465	1.004	
**Place of injury**	62			0.488	11			0.471
Outside	44	2.198	0.830		9	1.700	0.808	
Home	18	2.025	1.015		2	1.199	1.133	
**Etiology of injury**	62			0.780	11			0.491
Hit	36	2.214	0.826		9	1.522	0.905	
Object	18	2.038	1.011		2	2.000	0.000	
Assault	8	2.094	0.917		0	-	-	
**Period between cataract surgery and trauma (10 days to > 20 years)**								
< 6 months	8	1.306	0.985	0.0043	1	2		0.9372
6 months to 2 years	4	2.026	1.018		1	1.301		
> 2 years	31	2.107	0.897		6	1.516	0.817	
Missing	19	2.595	0.466		3	1.766	1.267	
Size of dehiscent phaco wound*								
< 4 mm					5	1.480	0.487	0.6633
4–6 mm					6	1.716	1.079	
**Clinical presentation**								
**Retinal detachment**	62			< 0.001	11			0.022
No	24	1.261	0.712		9	1.355	0.679	
Yes	38	2.708	0.371		2	2.750	0.212	
**Global deformity**	52			0.164	9			0.551
No	17	1.963	0.925		7	1.442	0.758	
Yes	35	2.317	0.807		2	1.848	1.063	
**IOL location**	62			0.010	11			0.206
In place	8	1.281	0.811		3	1.133	0.809	
Dislocated but inside globe	3	1.633	1.097		1	0.398	-	
Partially outside globe	2	2.600	0.424		3	2.067	0.802	
Completely outside globe	49	2.302	0.816		4	1.924	0.620	
**Vitreous hemorrhage**	62			0.203	11			0.712
No	9	1.800	0.853		6	1.700	0.968	
Yes	53	2.207	0.882		5	1.499	0.729	
**Hyphema**	62			0.510	11			0.167
No	5	2.400	0.797		5	1.219	0.802	
Yes	57	2.126	0.893		6	1.933	0.769	
**Initial vision (logMAR)**	62			0.005	11			0.256
25 percentile	23	2.19	0.284		9	2.089	0.428	
50 percentile	21	2.6	0		1	2.600	0	
75 percentile	18	2.900	0		1	2.900	0	
Ocular trauma score	62	29.98	12.60	< 0.001	11	39.55	11.27	0.008

Patients followed for < 1 month are excluded.

*SD* Standard deviation, *IOL* Intraocular lens.

*The size of dehiscent wound with prior phacoemulsification recorded at emergency room or operation room.

## Discussion

In this study, we investigated patients with traumatic cataract wound dehiscence between 2001 and 2015 and included 11 eyes with prior phacoemulsification. Previous studies with prior phacoemulsification have been mostly case reports. Only one time-series study was conducted by the Massachusetts Eye and Ear Infirmary group using data from 2000 to 2009 and it reported 7 cases of phacoemulsification wound^10^. This study had 6.3 cases of traumatic cataract wound dehiscence per year on average, and there were 5 cases per year in our hospital. The percentage of cases with phacoemulsification was 11% in Massachusetts study and 14% in ours. Our study showed that the type of prior cataract surgery was important in the visual outcome after traumatic cataract wound dehiscence, and RD together with OTS were the key risk factors for visual outcome. Low OTS, meaning poor ocular condition after injury, is a risk factor for lower final visual outcome in patients with prior ECCE or phacoemulsification.

Our study contributes to existing studies through the observation of patients over a relatively long period of 15 years within the same medical center; this allowed us to determine longitudinal trends in the variables of interest by controlling for time-fixed institutional effects. Moreover, we had a relatively large sample size in patients with prior ECCE or phacoemulsification, which allows us to do risk factors analysis for poor visual outcome in patients of these two groups. The longitudinal trend in our study revealed that patients with injured eyes before 2008 all had a prior ECCE instead of phacoemulsification. Cases involving patients with a history of phacoemulsification appeared almost every year since the first case in 2008. The phacoemulsification surgery leading to our first case of traumatic wound dehiscence was performed after the first US case was reported in 1999^6^. This is due to the earlier introduction of phacoemulsification in the United States. By 1995, phacoemulsification accounted for more than half of the cataract surgeries performed in the United States^5^. In Taiwan, phacoemulsification was first introduced in 1992, and our hospital was one of the first medical centers to adopt this procedure^15^. The phacoemulsification penetration rate at our hospital increased rapidly from 6.6% in 1993 to 23.6% in 1994^15^. In Taiwan, phacoemulsification has been the most commonly performed form of cataract surgery. For example, 93.3% of the 342 cataract surgeries performed at our hospital in October 2008 were phacoemulsification surgeries. The remaining surgeries were ECCE, lensectomy–vitrectomy, and lens aspiration (3.8%, 2.6%, and 0.3%, respectively). Although wound dehiscence is less commonly observed with phacoemulsification than with ECCE, phacoemulsification-associated wound dehiscence should not be overlooked in cases of blunt ocular injury because phacoemulsification with IOL implantation has become the most frequently performed cataract procedure in developed countries^4,5^.

The most common mechanism of traumatic cataract wound dehiscence in all our patients was fall (63%), which is similar to a previous study^10^. It was also fall (81%) that mainly caused the traumatic cataract wound dehiscence in patients with prior phacoemulsification. The most common place where the accident took place was home in patients both with prior ECCE or phacoemulsification. Studies have indicated that falls constitute the main cause of open globe injury in older adults^16,17^. Falls are one of the main causes of morbidity and disability in the elderly population. One-third of community-dwelling people aged over 75 years have at least one fall episode each year^18^. To prevent the devastating effects on visual outcome after traumatic cataract wound dehiscence, fall prevention in the older population is essential^19^. Seventy-five percent of the patients included in our study had a history of one or more chronic systemic diseases, and it is not uncommon for older patients as in our study to have these health conditions^20,21^. Although a comorbidity may not itself lead to a higher likelihood of traumatic eye injuries, it may place more burden on patients in terms of care management when such injuries occur, thus significantly reducing the quality of life.

We found that patients with traumatic wound dehiscence with prior phacoemulsification had better final vision than those with ECCE, and this finding is similar to the Massachusetts study^10^. In the Massachusetts study, the median final VA was 0.48 in the phacoemulsification group, and no eyes developed retinal detachment. In our study, the median LogMAR of final VA was 2.0 in the phacoemulsification group, and 2 eyes were complicated with retinal detachment associated with severe hemorrhagic suparachoroidal detachment. The mean follow-up duration in the phacoemulsification group in that study (24 days) was shorter than in ours (2 to 108 months). The worse visual outcome of patients with prior phacoemulsification in our study may be due to longer follow-up periods and possible stronger traumatic force with inadequate fall prevention in our patients. Because traumatic wound dehiscence with prior phacoemulsification only occurred after 2008 in our study, we also compared the final VA in all patients with traumatic cataract wound dehiscence before and after (including) 2008 to determine whether the phacoemulsification group had better visual outcomes with time, but we did not find evidence for this speculation (*P* = 0.85). We also noted that eyes with prior phacoemulsification had a lower percentage of globe deformity compared with those with prior ECCE, as suggested by imaging studies (*P* = 0.02). All the patients in our study with blunt open-globe injury had cataract wound dehiscence in both ECCE and phacoemulsification groups. This result is similar to previous studies^2,3,6–10^. All these results indicate that cataract wounds create a weak area in the eye globe. Smaller cataract wounds preserve globe integrity better and thus provide better protection against an external force. Other risk factors associated with worse final VA were retinal detachment, worse initial VA, and IOL dislocation. Compared with patients with globe rupture injury or open globe injury in the study of Stryjewski et al., our patients with traumatic wound dehiscence from cataract surgery had a higher incidence rate and an earlier onset of RD^22^. RD was noted in 59% of our patients, with most instances presented at the initial visit. In their study, RD was a complication in 29% of patients with open globe injury and in 42% of patients with globe rupture injury. RD occurred within 24 h of open globe injury in only 27% of cases^22^. Retinal detachment and initial VA were also reported as risk factors of poor final VA in studies of open-globe injuries^23^. OTS includes these major risk factors and is a good overall predictor for final VA in our study.

This study had some limitations. First, this was a retrospective study, and data of some variables were missing. Thus, we had to exclude patients with missing data in some analyses. Second, the follow-up period was not similar for all patients in our study, and some were followed up for < 6 months. Certain vision-threatening complications could not develop in this short period of time. However, > 80% of patients in the ECCE group and > 70% in the phacoemulsification group were followed up for more than 6 months, so the short follow-up time for the small number of patients should not be a serious concern. Third, the number of traumatic wound dehiscence cases with prior phacoemulsification is still small even though this study has the largest series reported so far.

Traumatic cataract wound dehiscence has continued to be an etiology of vision-threatening open-globe injury in our hospital. Most of these patients had prior ECCE, but after phacoemulsification had become a popular choice for cataract treatment, wound dehiscence was noted in this group of patients, and visual outcome can be poor once it was complicated with retinal detachment and presented with a low ocular trauma score. Ophthalmologists in the emergency room should carefully examine every case of blunt ocular injury for cataract wound dehiscence regardless of whether prior ECCE or phacoemulsification was performed. However, patients with prior phacoemulsification had better vision outcomes than those with prior ECCE. Phacoemulsification with a smaller cataract wound is a better surgical option than ECCE for blunt trauma protection.
